# Tumoral Melanosis Associated with Pembrolizumab-Treated Metastatic Melanoma

**DOI:** 10.7759/cureus.1026

**Published:** 2017-02-13

**Authors:** Omar Bari, Philip R Cohen

**Affiliations:** 1 School of Medicine, University of California, San Diego; 2 Department of Dermatology, University of California, San Diego

**Keywords:** antibody, immunotherapy, melanoma, melanosis, metastasis, pembrolizumab, tumoral

## Abstract

Tumoral melanosis is a form of completely regressed melanoma that usually presents as darkly pigmented lesions suspicious for malignant melanoma. Histology reveals dense dermal and subcutaneous infiltration of melanophages. Pembrolizumab is an antibody directed against programmed death receptor-1 (PD1) and is frontline treatment for advanced melanoma. An 81-year-old man with metastatic melanoma treated with pembrolizumab who developed tumoral melanosis at previous sites of metastases is described. The PubMed database was searched with the key words: antibody, immunotherapy, melanoma, melanosis, metastasis, pembrolizumab, and tumoral. The papers generated by the search and their references were reviewed. The patient was initially diagnosed with lentigo maligna melanoma on the left cheek three years earlier, and he was treated with wide local excision. The patient was subsequently diagnosed with epidermotropic metastatic malignant melanoma on the left parietal scalp 14 months later and was treated with wide local excision. Three months later, the patient was found to have metastatic melanoma in the same area of the scalp and was started on pembrolizumab immunotherapy. The patient was diagnosed with tumoral melanosis in the site of previous metastases nine months later. The patient remained free of disease 13 months after starting pembrolizumab. Tumoral melanosis may mimic malignant melanoma; hence a workup, including skin biopsy, should be undertaken. Extensive tumoral melanosis has been reported with ipilimumab, and we add a case following treatment with pembrolizumab. Additional cases of tumoral melanosis may present since immunotherapy has become frontline therapy for advanced melanoma.

## Introduction

Tumoral melanosis is a rare presentation of completely regressed melanoma that presents as darkly pigmented lesions, which are clinically suspicious for melanoma. Histology reveals dense dermal and subcutaneous infiltration of heavily pigmented monocytes, referred to as melanophages [1]. Pembrolizumab is a humanized monoclonal antibody directed against programmed death receptor-1 (PD1) that is approved as frontline treatment of advanced melanoma [2]. We describe the clinical features of a man with tumoral melanosis whose metastatic melanoma was treated with pembrolizumab, and we review the literature of tumoral melanosis.

## Case presentation

**Figure 1:**
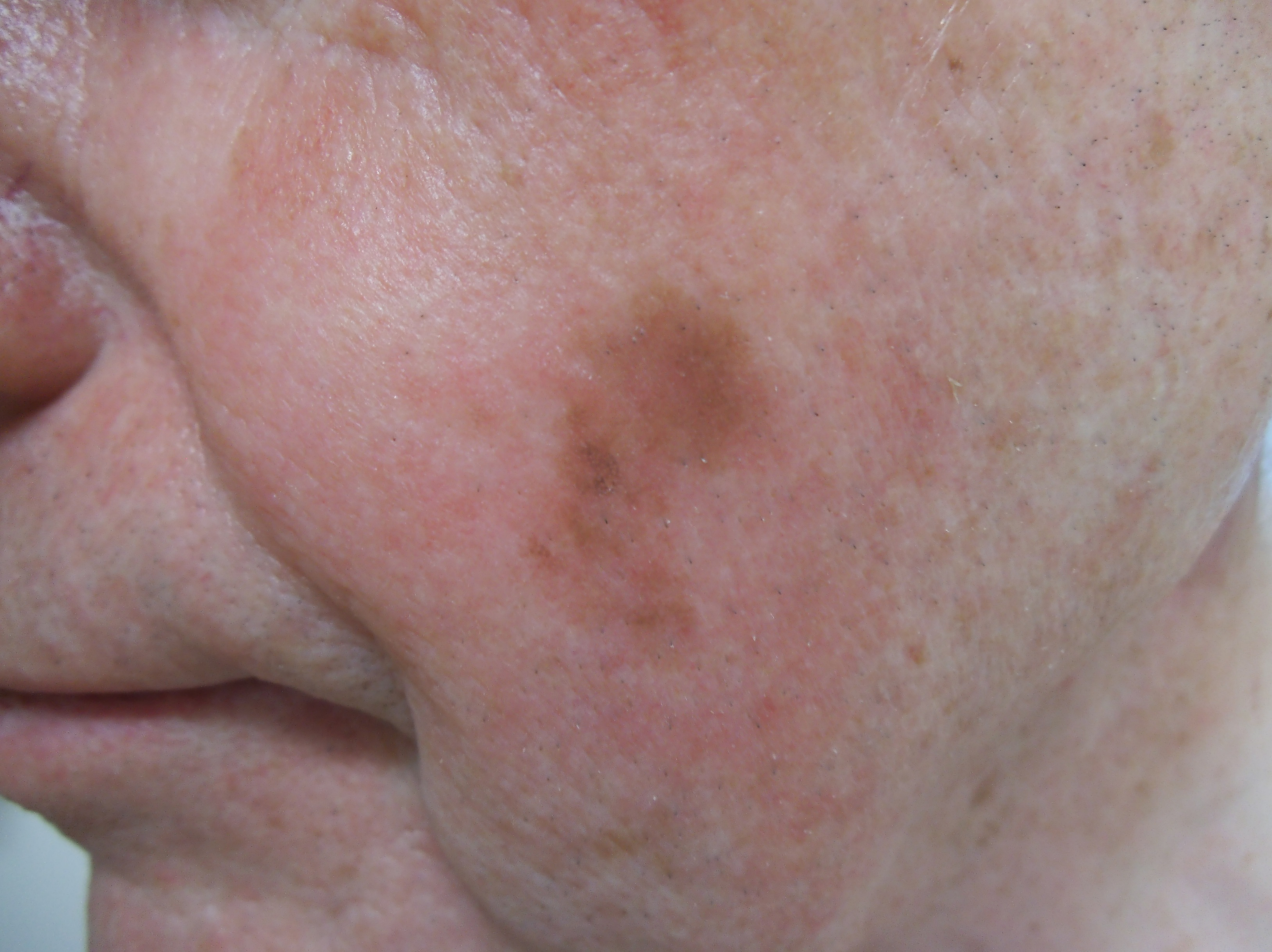
Lentigo maligna melanoma on the left cheek. A 2 x 1 cm brown patch is observed on the patient’s left cheek.

**Figure 2:**
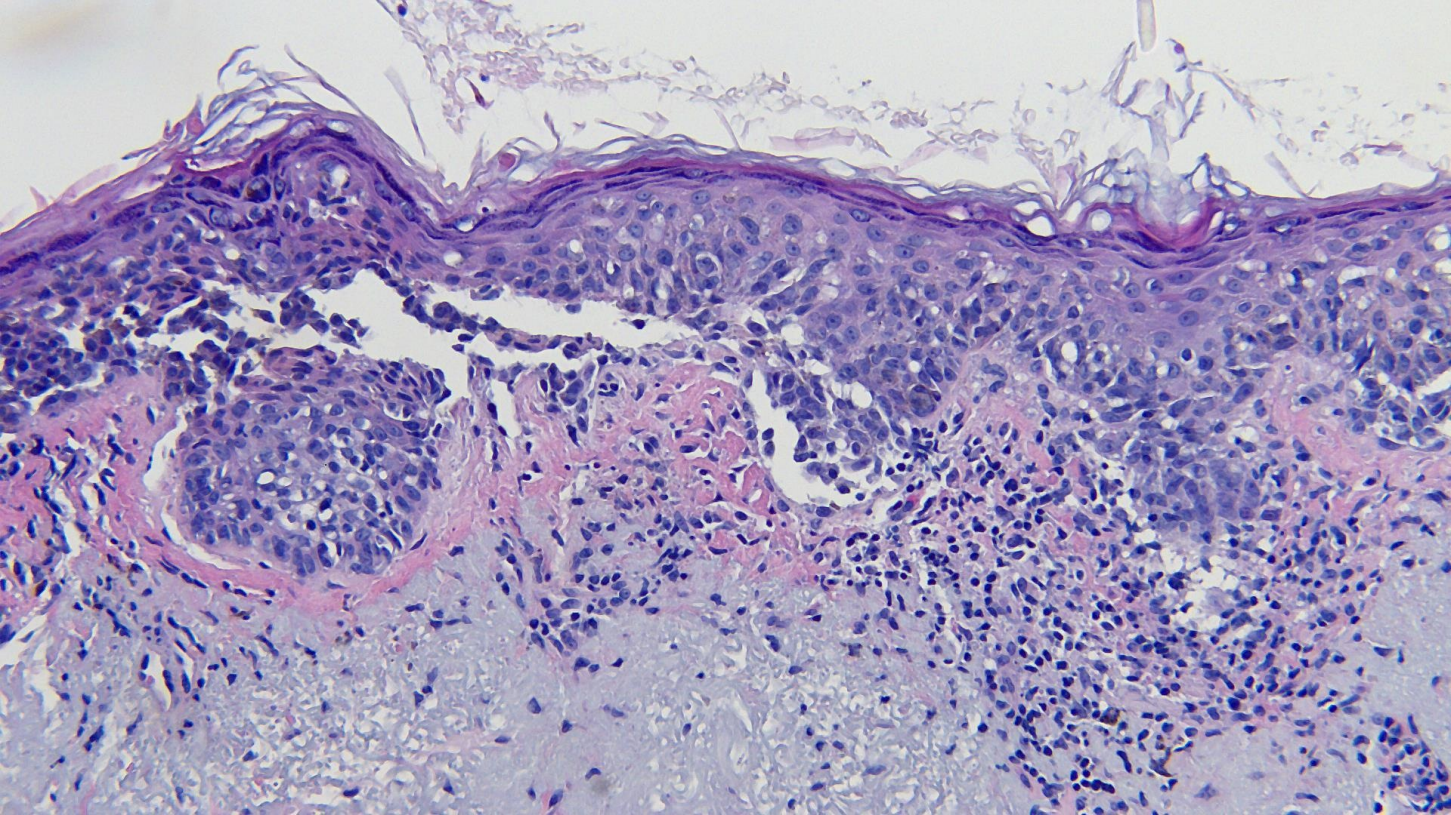
Microscopic examination of the lentigo maligna melanoma biopsy. Atypical melanocytes are seen along the dermal-epidermal junction. There is also extension of the tumor cells down adnexal structures (hematoxylin and eosin; x = 20).

**Figure 3:**
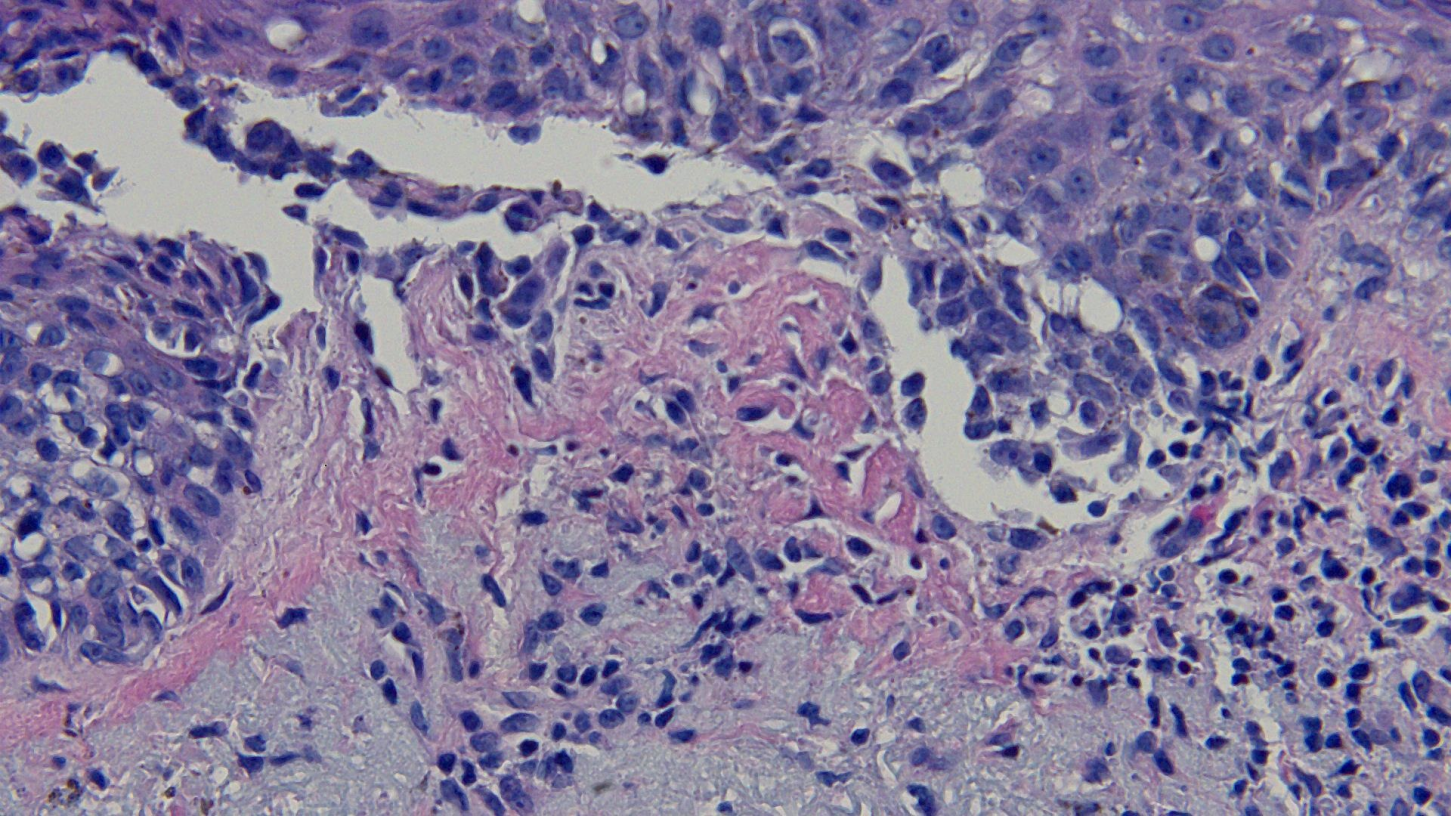
Closer magnification of the microscopic examination of the lentigo maligna melanoma biopsy. Closer view of the atypical melanocytes along the dermal-epidermal junction (hematoxylin and eosin; x = 40).

**Figure 4:**
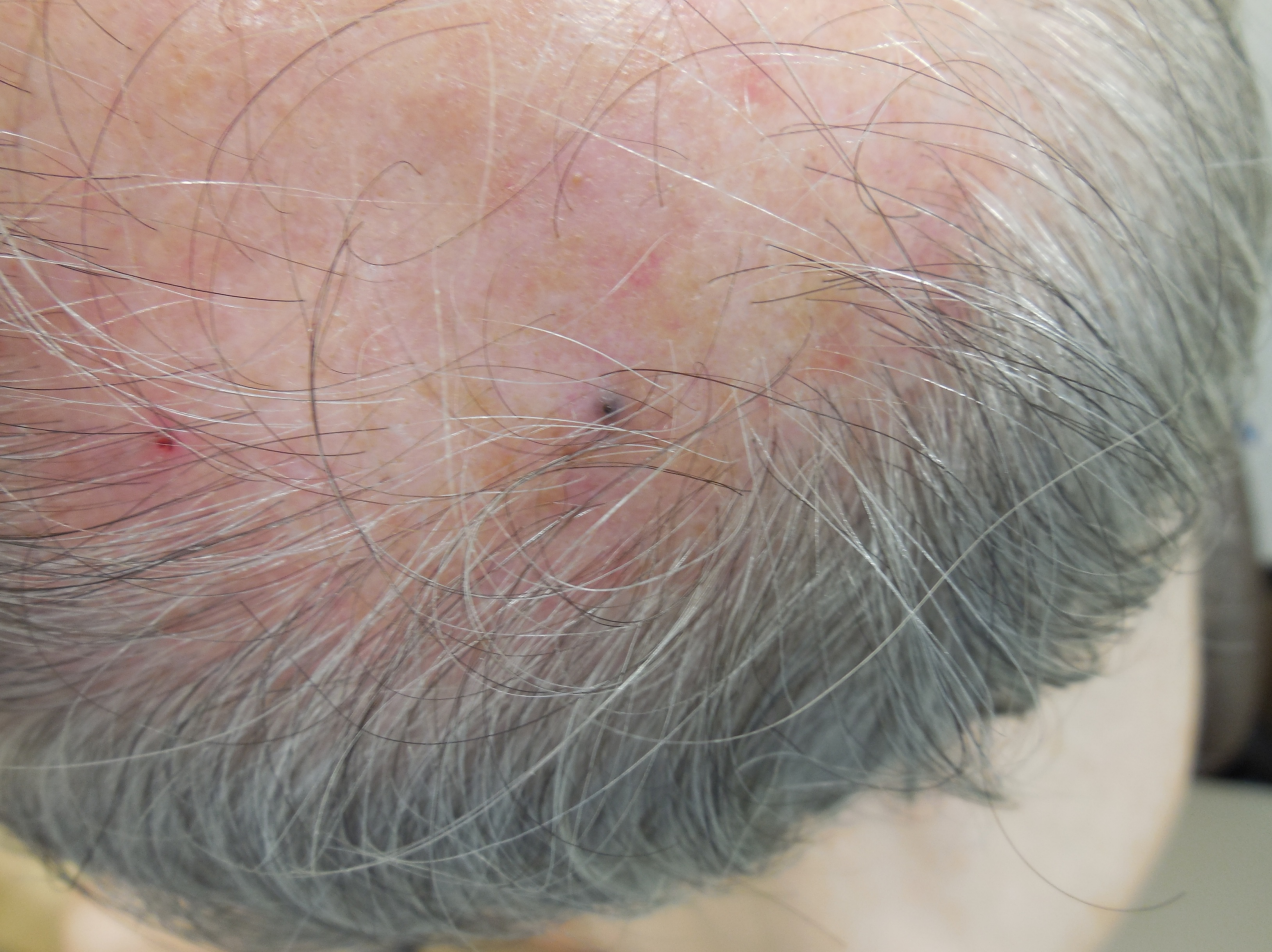
Epidermotropic metastatic malignant melanoma on the left parietal scalp. Metastatic malignant melanoma presents here as a 2 mm black papule on the left parietal scalp, mimicking an open comedone.

**Figure 5:**
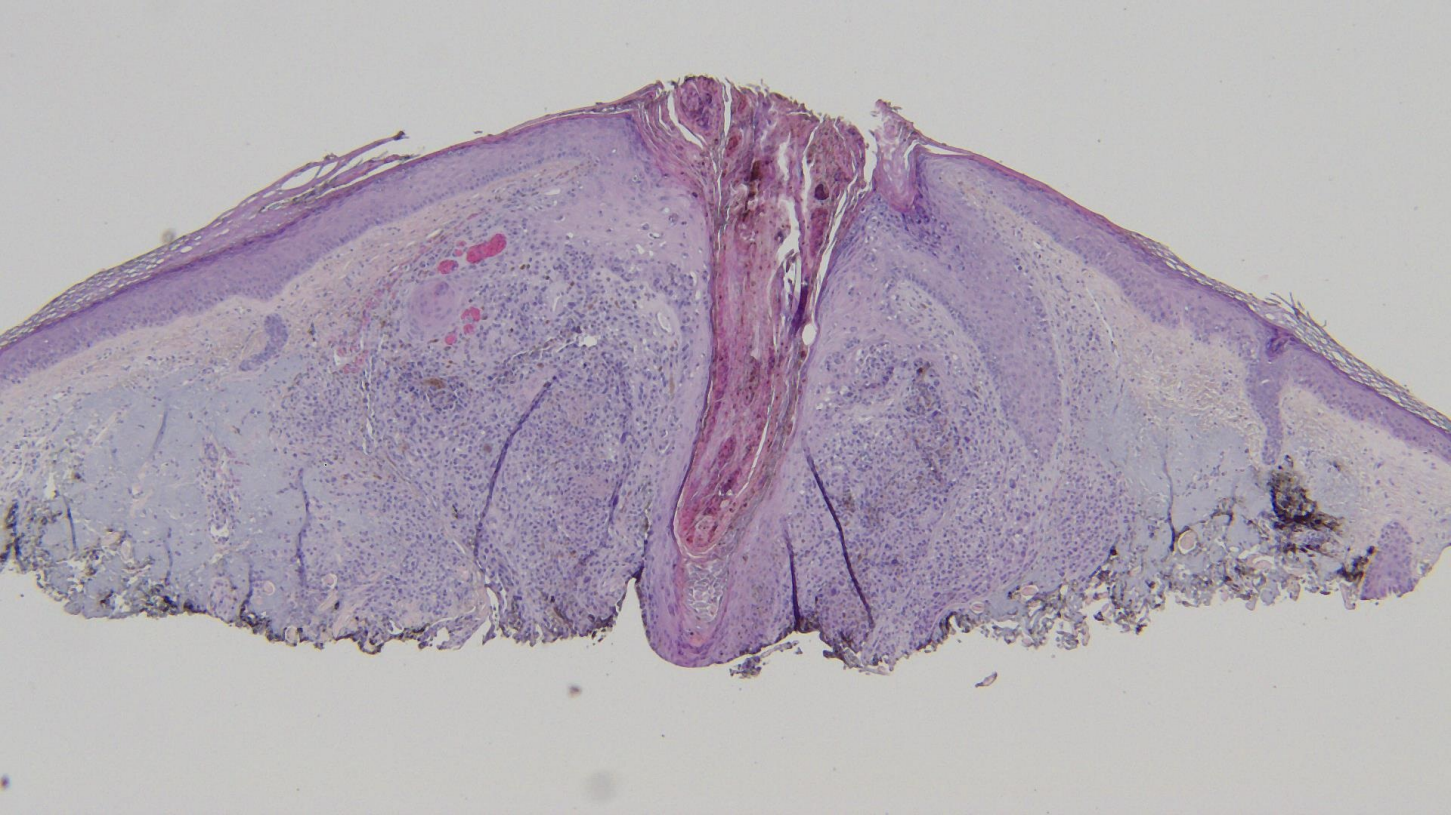
Microscopic examination of the initial shave biopsy of the epidermotropic metastatic malignant melanoma. Surrounding a central follicle, a dense infiltrate of melanocytes in the dermis and epidermis is seen (hematoxylin and eosin; x = 4).

An 81-year-old man with a history of nonmelanoma skin cancer, currently being treated with pembrolizumab for metastatic melanoma, presented for evaluation three blue patches on his left lateral posterior scalp. He provided a signed photo consent for clinical images to be used. Three years earlier, he presented with a 2 x 1 cm brown patch on his left cheek (Figure 1). Biopsy showed a proliferation of atypical melanocytes along the dermal-epidermal junction. The melanocytes demonstrated moderate cytologic atypia, and they extended down adnexal structures. Small nests of atypical melanocytes were also seen in the superficial papillary dermis associated with lymphocytic inflammation (Figures 2-5). These findings were diagnostic of lentigo maligna melanoma with a Breslow depth 0.1 mm and a Clark level II. The patient was treated with wide local excision.

Cutaneous surveillance, 14 months later, revealed a 2 mm black papule on the left parietal scalp (Figure 4). Shave biopsy showed a follicle in the center of the specimen with a dense infiltrate of melanocytes in the dermis and extending into the overlying epidermis (Figure 5). Examination of the site, after removing the shave biopsy specimen, showed pigment at the base of the biopsy.

**Figure 6:**
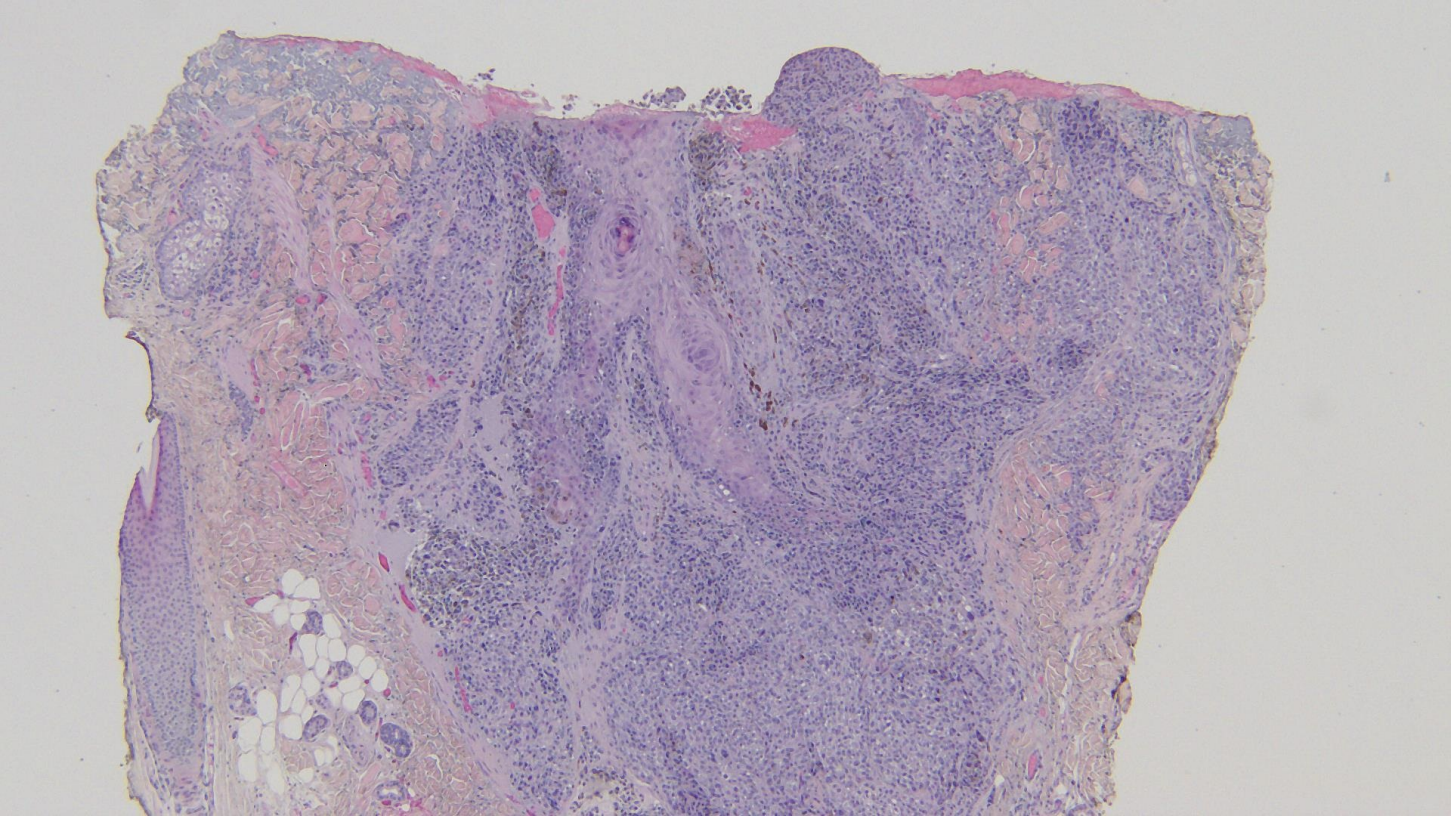
Distant view of the subsequent punch biopsy to evaluate the residual pigment observed clinically in the center of the biopsy site after the shave biopsy was performed. There is a nodular configuration of atypical melanocytes in the papillary and reticular dermis with small areas of junctional involvement and folliculotropism (hematoxylin and eosin; x = 4).

**Figure 7:**
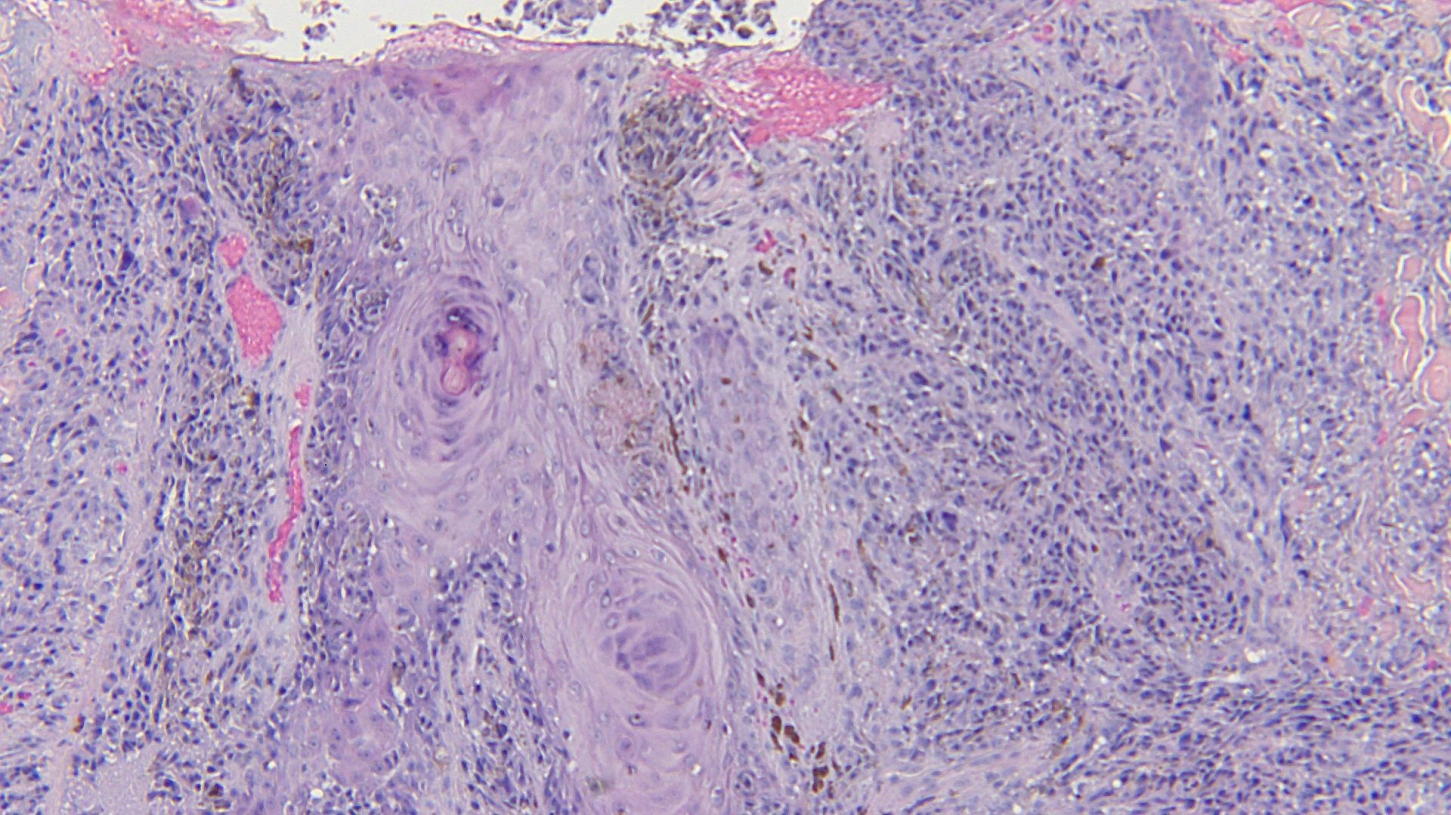
Closer magnification of the microscopic examination of the punch biopsy of the epidermotropic metastatic malignant melanoma. Atypical tumor melanocytes fill the dermis and extend into the follicular epithelium and overlying epidermis (hematoxylin and eosin; x = 20).

**Figure 8:**
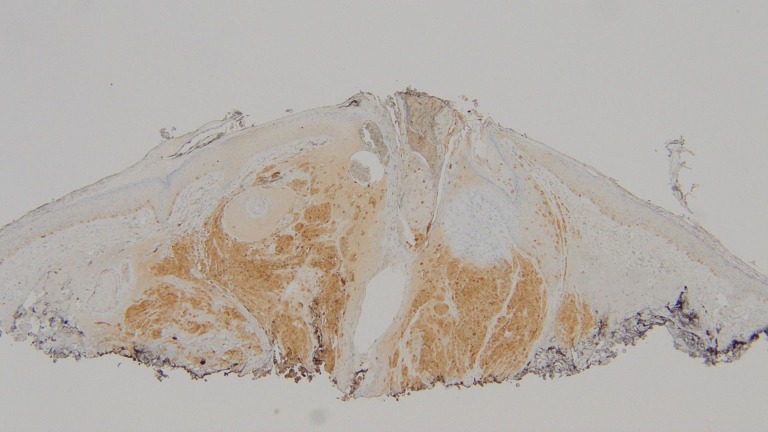
S100 stain of the shave biopsy of the epidermotropic metastatic malignant melanoma. The tumor cells demonstrate positive S100 staining, confirming the identification of the neoplasm as a melanoma (hematoxylin and eosin; x = 4).

A punch biopsy was then taken to remove the pigmented area; pathology showed an aggregate of atypical melanocytes in a nodular configuration in the papillary and reticular dermis with small areas of junctional involvement and folliculotropism (Figures 6-7). The tumor cells demonstrated positive staining with S100 (Figure 8). These findings were consistent with epidermotropic metastatic malignant melanoma. The patient was not only referred to a head and neck oncologic surgeon, but he was also presented at a multidisciplinary tumor board. Wide local excision with flap repair was recommended and performed.

**Figure 9:**
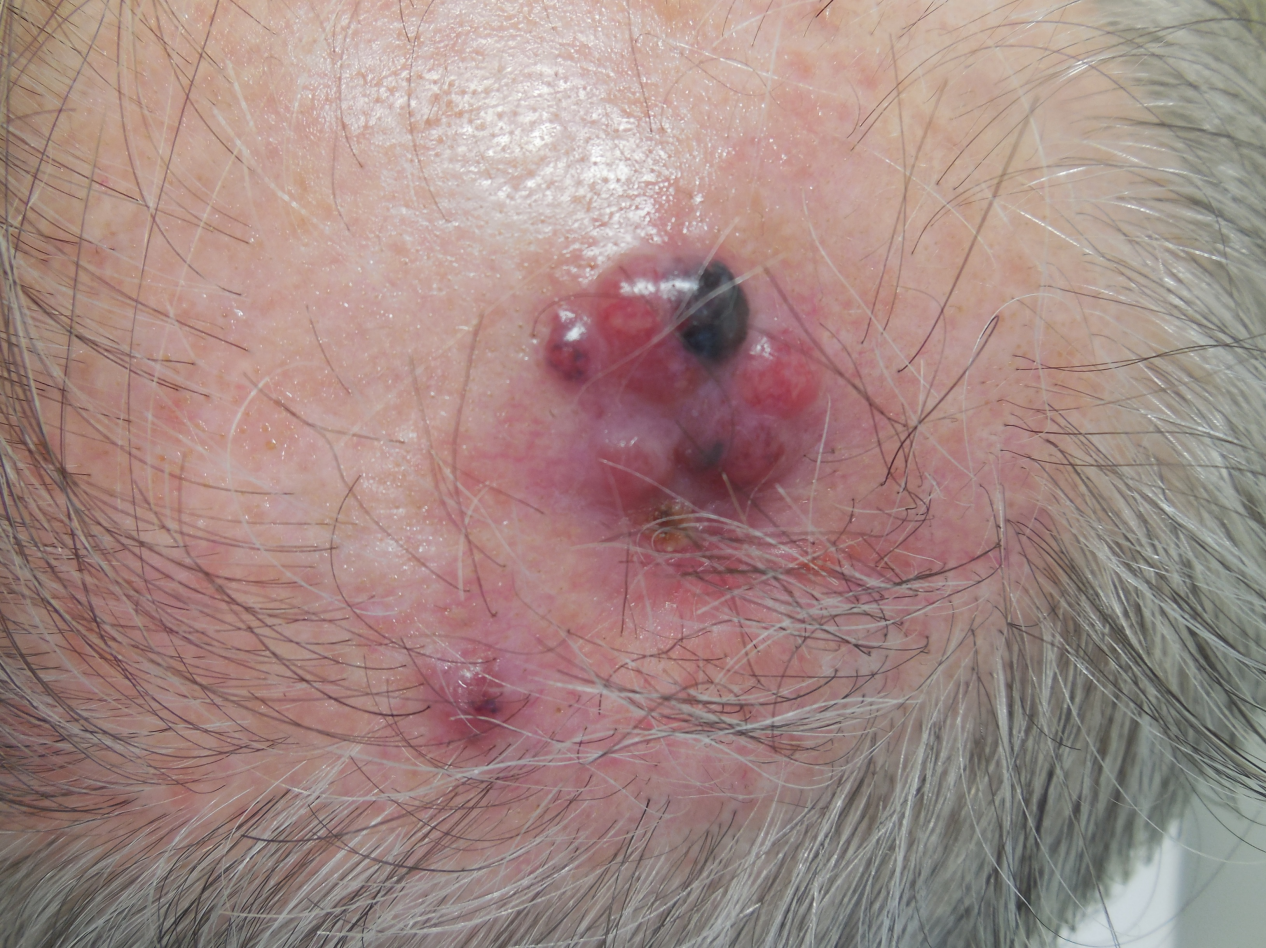
Metastatic melanoma on the left parietal scalp. Multiple red nodules, ranging from 6 to 8 mm in diameter, with a focal black patch, are observed.

**Figure 10:**
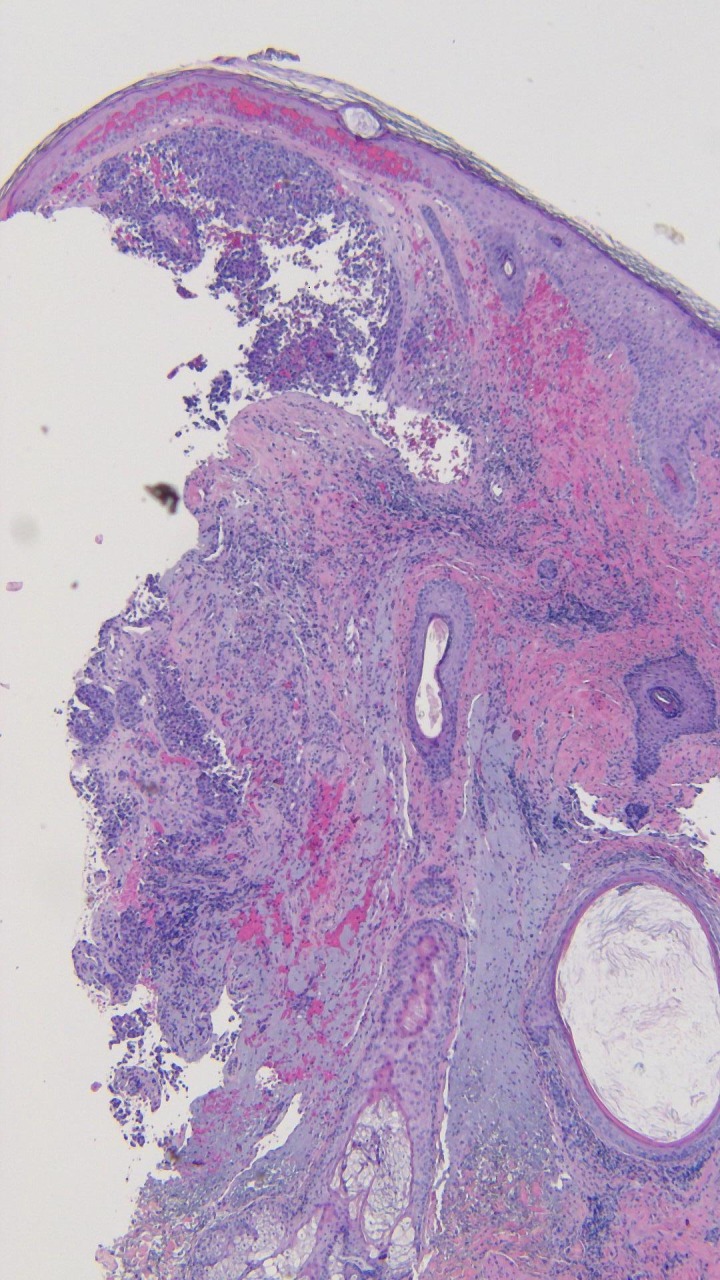
Microscopic examination of the punch biopsy of the metastatic melanoma on the left parietal scalp. Atypical melanocytes are seen in a nodular configuration in the papillary dermis and extend deeply into the reticular dermis (hematoxylin and eosin; x = 4).

**Figure 11:**
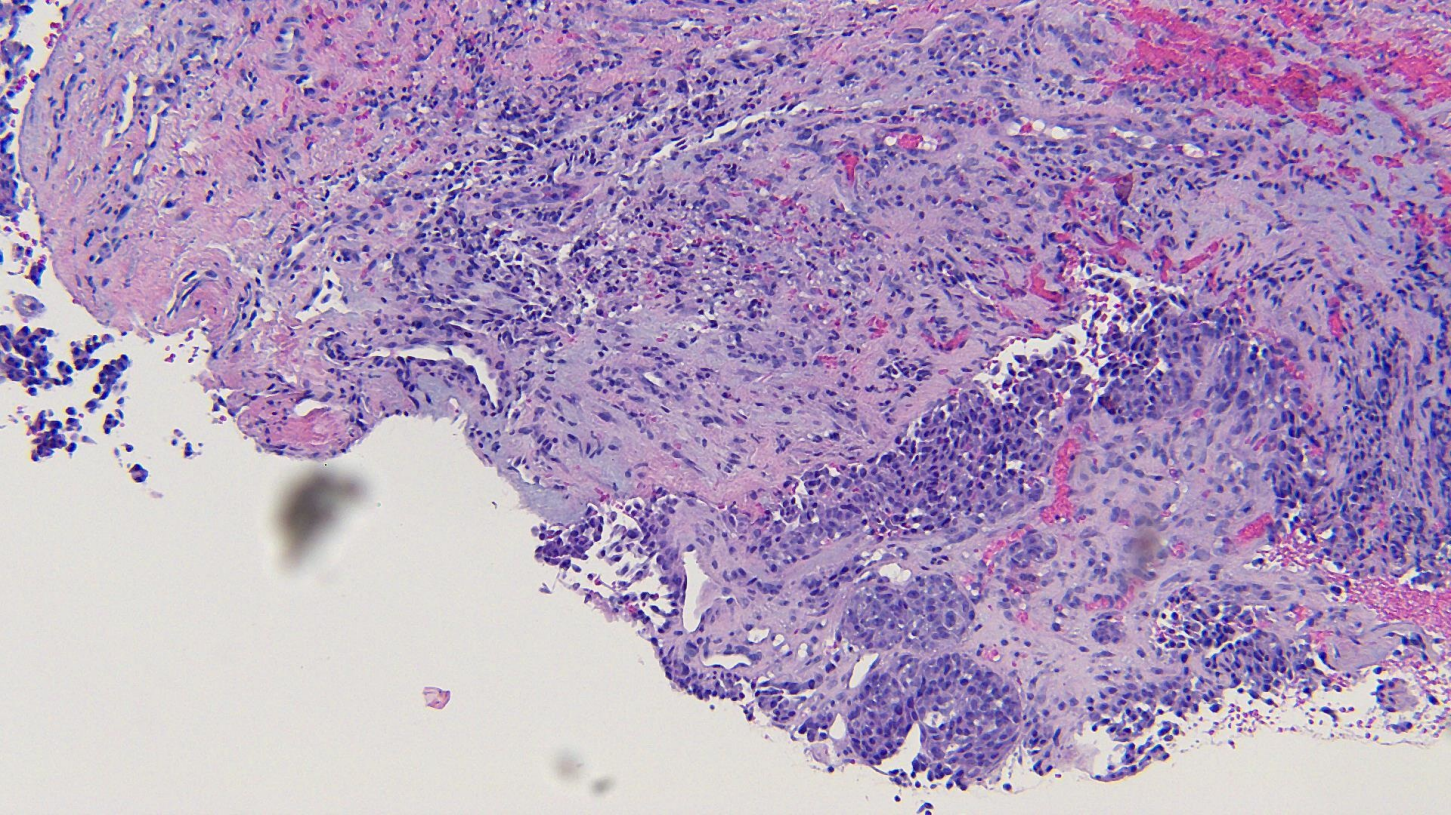
Closer magnification of the microscopic examination of the punch biopsy of the metastatic melanoma on the left parietal scalp. Atypical melanocytes in the deep dermis are seen in this closer view (hematoxylin and eosin; x = 20).

The patient presented with multiple red nodules, ranging from 6 to 8 mm in diameter, in the same left parietal scalp region within three months (Figure 9). A punch biopsy showed an aggregate of atypical melanocytes in a nodular configuration in the papillary and reticular dermis (Figures 10-11). S100 stained positive to highlight cells comprising the proliferation. These findings established a diagnosis of metastatic malignant melanoma.

The patient received computerized tomography (CT) scans of the head, neck, chest, abdomen, and pelvis that confirmed the scalp lesions; however, there were no other sites of metastatic disease. Pembrolizumab therapy, dosed at 200 mg, was initiated with infusions scheduled every three weeks. A successful clinical response, with diminishing lesion size, was evident within two months of starting therapy.

**Figure 12:**
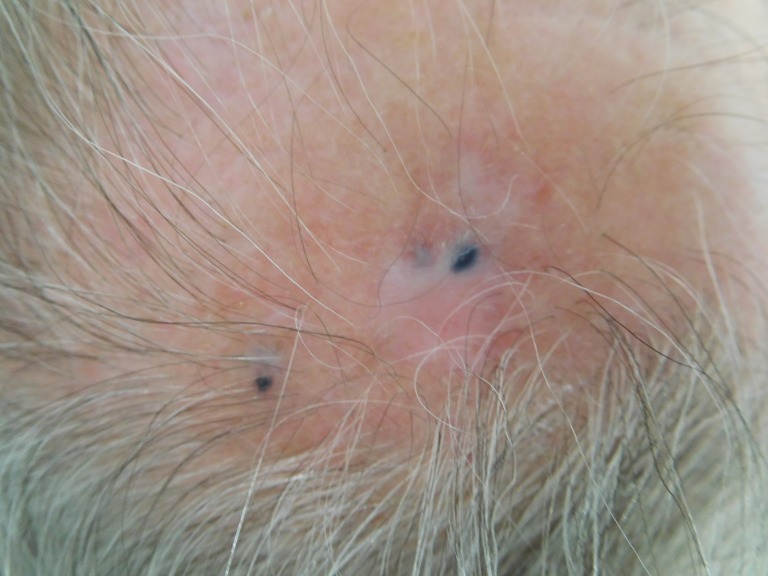
Tumoral melanosis on the left parietal scalp. Three blue patches, ranging from 2 to 4 mm in diameter are observed.

**Figure 13:**
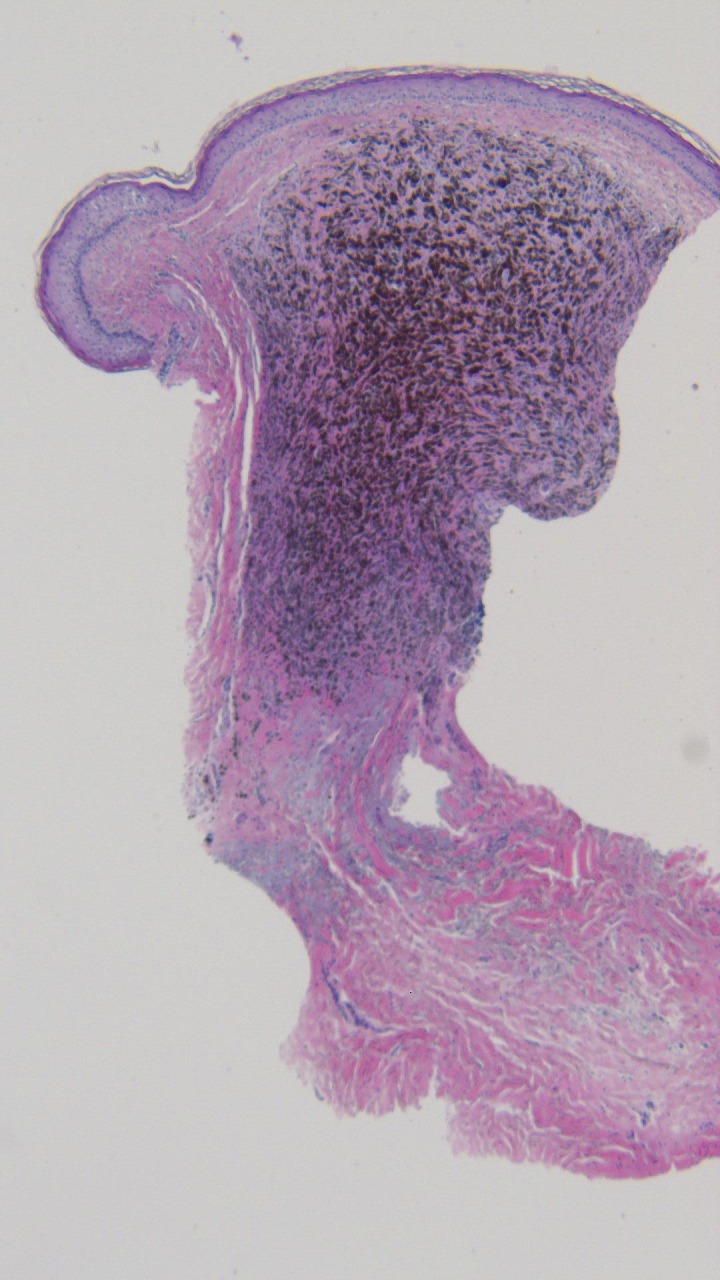
Microscopic examination of the punch biopsy of the tumoral melanosis on the left parietal scalp. Dense collections of melanophages are seen in a nodular aggregate; tumor melanocytes are not observed (hematoxylin and eosin; x = 4).

**Figure 14:**
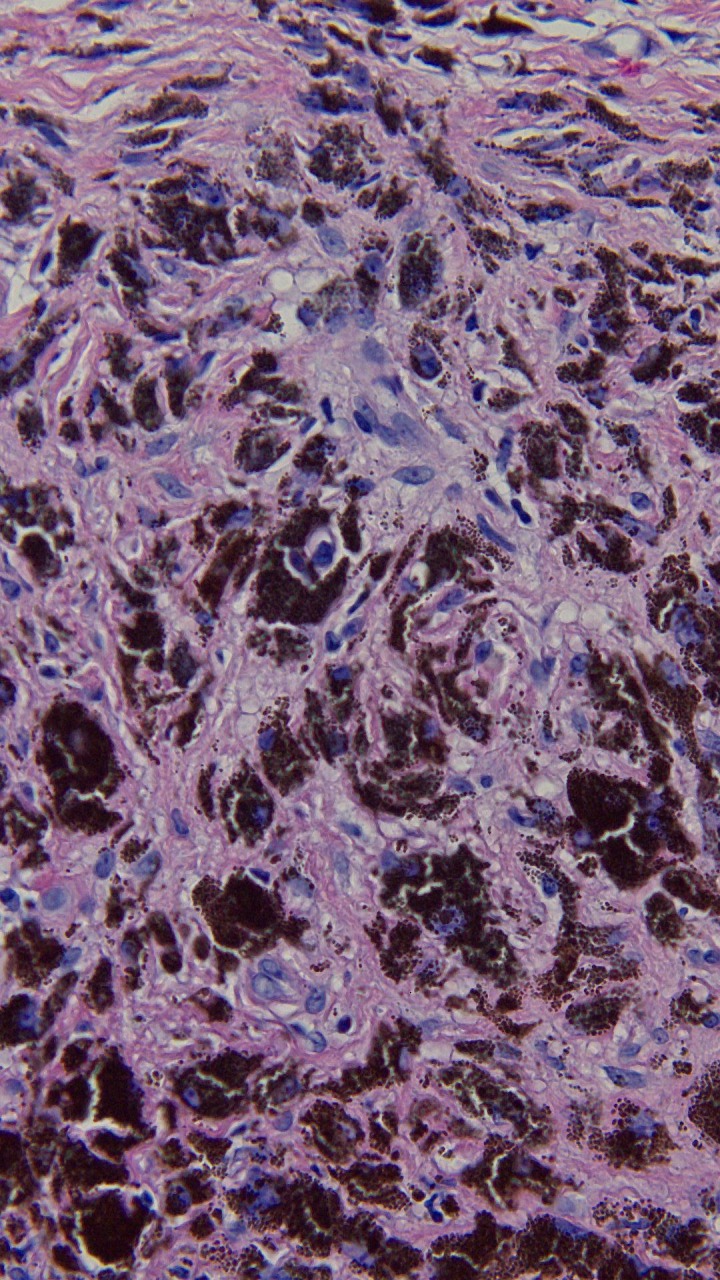
Closer magnification of the microscopic examination of the punch biopsy of the tumoral melanosis on the left parietal scalp. The superficial and deep reticular dermis are filled with melanophages, and residual melanoma is absent (hematoxylin and eosin; x = 40).

Three blue macules were seen at the site of prior tumor metastases three months after starting pembrolizumab. Six months later, after 15 cycles of pembrolizumab, the three blue macules still remained on the left parietal scalp (Figure 12). A punch biopsy was performed and pathology revealed dense collections of melanophages in a nodular aggregate in the dermis; there was no residual melanoma (Figures 13-14). Correlation of pathology and clinical features established the diagnosis of tumoral melanosis. The patient has been maintained on pembrolizumab with no evidence of recurrent melanoma.

## Discussion

Pembrolizumab interrupts tumor-induced immune suppression in melanoma by blocking the PD-1 receptor [2]. PD-1 receptors, once bound to their ligands, negatively regulate B-cell and T-cell function; this allows the tumor to thwart immune surveillance [2]. Pembrolizumab is approved as frontline treatment of advanced melanoma; it aims to restore the cytotoxic function of lymphocytes that infiltrate the tumor [2]. Adverse effects most commonly associated with this medication include diarrhea, fatigue, pruritus, and rash; anemia and pneumonitis have also been reported [2].

Tumoral melanosis is an atypical presentation of completely regressed melanoma [1]. Including our patient, 13 cases of melanoma-associated tumoral melanosis have been reported (Table 1) [3-10]. Of the nine cases with gender information provided, there are five men and four women. Patient age ranges from 32 years to the late 80s. The types of melanoma included epidermotropic metastatic melanoma (one case), lentigo maligna melanoma (two cases), malignant melanoma (two cases), melanoma with in-transit metastases (one case), metastatic melanoma (one case), and superficial spreading melanoma (two cases).

**Table 1:** Characteristics of tumoral melanosis in patients with melanoma [a]. Abbreviations: A, age (in years); C, case; Ca, Caucasian; CR, case report; CT, computerized tomography scan; DF, disease-free; DFD, died from disease; FU, follow-up; Hx, history; IT, in-transit melanoma; L, left; LM, lentigo maligna; LN, lymph node; Loc, location; LT, long-term; M, man; Mel, melanoma; MM, metastatic melanoma; Mo, Moroccan; ND, not detected; NR, not reported; R, right; Ra, race; Re, reference; S, sex; SSM, superficially spreading melanoma; TM, tumoral melanosis; W, woman; WLE, wide local excision. [a] Cases 10-13 included two individuals with metastatic melanoma and one patient with in-transit melanoma; no additional details were provided [10].

C	A Ra S	Clinical presentation	Loc	Mel status	Known mel hx	Comments	LT FU	Re
1	32 Mo W	12 2-cm blue-black macules	Trunk and legs	IT	No	Macule biopsy was consistent with TM. Three pea-sized subcutaneous nodules lacking surface pigmentary changes were also found; biopsy revealed atypical melanocytes with pleomorphic nuclei. A small inguinal LN was biopsied and revealed no neoplastic cells.	DF after three years	3
2	36 NR M	Palpable mass	L axilla	MM	No	4.5 cm L axillary LN was excised and revealed MM. Subsequently, 24 axillary LN were removed; one LN was positive for MM and 13 LN showed TM.	NR	4
3	58 NR M	Black macules and papules	Scalp	ND	No	4 cm area of dark confluent macules and papules on scalp was excised along with two cervical LN, revealing TM.	DF after one year	5
4	66 NR W	Pigmented macule	L thigh	MM	No	Presented with 8 cm L inguinal mass and 6 x 10 mm pigmented macule on L thigh; thigh lesion was present for five years. Macule biopsy revealed TM, though L inguinal mass revealed MM. CT showed disseminated disease.	DFD four months later	6
5	75 Ca M	Blue-black macule	Upper back	ND	Yes	Macule developed over four months. History of mel and LM on the back, though these lesions were distant from TM lesion site.	DF after eight months	7
6	75 NR W	Palpable mass	R groin	ND	Yes	Presented nine years after excision of 1 mm thick mel on R leg. Staging CT showed 10 mm lymph node in R groin. Lymphadenectomy revealed six nodes negative for disease.	NR	8
7	79 Ca M	Multiple purple-black papules and nodules	L elbow	IT treated with ipilimumab with no active disease	Yes	Initially diagnosed with SSM on L forearm and treated with WLE. Two months later patient had many small dark papules near surgical scar and diagnosed with IT metastases; patient started on ipilimumab. Two months later patient diagnosed with TM that developed within and adjacent to surgical site.	DF one year later	1
8	81 Ca M	Three blue patches	L parietal scalp	MM treated with pembrolizumab with no active disease	Yes	Initially diagnosed with LM on L cheek three years prior and treated with WLE. 14 months later patient diagnosed with epidermotropic metastatic mel on L parietal scalp and treated with WLE. Three months later patient diagnosed with MM in same scalp region and started on pembrolizumab. Nine months later after 15 cycles of pembrolizumab, patient diagnosed with TM at site of metastases.	DF 13 months later	CR
9	80s NR W	Blue-black nodule	L scapular back	MM	Yes	Five-month history of 2.8 cm pigmented nodule. Diagnosed with SSM on R forearm 11 years prior.	DFD months later	9

The clinical manifestation of tumoral melanosis is often a darkly pigmented cutaneous lesion that is suspicious for melanoma. The 13 cases reviewed included blue-black macules, papules, patches, and nodules. These lesions were observed at various skin locations. However, two patients lacked skin lesions; they instead presented with palpable masses. The clinical differential diagnosis for tumoral melanosis includes deep penetrating nevus, epithelioid blue nevus, epithelioid cyst, malignant blue nevus, malignant melanoma, pigmented basal cell carcinoma, and squamous cell carcinoma [1,9].

The histopathology of tumoral melanosis, as seen in our patient, is characterized by dense dermal and subcutaneous infiltrates of melanophages without melanocytes [1,5]. Melanophages may extend to lymph nodes [4-5]. These melanin-laden macrophages stain positive for CD68 and negative for HMB45, Melan A, S100, and SOX10 [4,8].

The prognosis of tumoral melanosis is variable as it hinges on the degree of regression of the underlying tumor [9]. No guidelines on treatment exist, but the potential for underlying melanoma with distant metastases behooves a complete work-up [1]. In our review of tumoral melanosis, five cases were concurrently diagnosed with underlying metastatic melanoma; for those patients with follow-up reported, death from disease within months of this diagnosis was common. In contrast, three patients had no detected underlying disease at all. Two patients were found to have in-transit metastases; the patient with long-term follow-up reported was disease-free after three years.

Two individuals, one being our patient, were on immunotherapy at the time of tumoral melanosis diagnosis. A 79-year-old man with melanoma with in-transit metastases was being treated with ipilimumab; this medication is a checkpoint inhibitor that blocks the cytotoxic T-lymphocyte-associated antigen-4 receptor in order to enhance cytotoxic T-lymphocyte activity. Our patient with metastatic melanoma was being treated with pembrolizumab. Both men were disease-free at one-year follow-up. 

## Conclusions

Tumoral melanosis appears to afflict both men and women with a predilection for those over the age of 60 years. Often patients with tumoral melanosis carry a known history of melanoma; lentigo maligna melanoma and superficial spreading melanoma were the most common specific types of melanoma in those with a known melanoma history. Prognosis is variable, depending on the status of the underlying tumor; those individuals with metastatic disease fare poorly, while those with no active disease or in-transit metastases tend to be disease-free with longer-term follow-up. However, the sample size of tumoral melanosis cases is limited, and therefore it is difficult to make broad conclusions on prognosis. Including our patient, immunotherapy led to favorable results in two patients with metastatic melanoma. As the melanoma tumor burden was reduced, tumoral melanosis resulted in its place; this implies that immunotherapy with pembrolizumab and ipilimumab may contribute to a tendency for development of tumoral melanosis. As the use of these novel agents increases, additional cases of tumoral melanosis may occur as a result of tumor response to immunotherapy.
